# Safety and Tolerability of Lacosamide in Patients With Epilepsy: A Systematic Review and Meta-Analysis

**DOI:** 10.3389/fphar.2021.694381

**Published:** 2021-09-20

**Authors:** Chunsong Yang, Yuxuan Peng, Lingli Zhang, Li Zhao

**Affiliations:** ^1^ Department of Pharmacy, Evidence-Based Pharmacy Center, West China Second Hospital, Sichuan University, Chengdu, China; ^2^ Key Laboratory of Birth Defects and Related Diseases of Women and Children (Sichuan University), Ministry of Education, Chengdu, China; ^3^ West China School of Pharmacy, Sichuan University, Chengdu, China; ^4^ Department of Health Policy and Management, West China School of Public Health/West China Fourth Hospital, Sichuan University, Chengdu, China

**Keywords:** lacosamide, epilepsy, safety, systematic review, meta-analysis

## Abstract

**Background:** As a third-generation antiseizure medication (ASM), lacosamide (LCM) is recommended worldwide for patients with epilepsy. We aimed to provide more conclusive evidence for the safety and tolerability of LCM in patients with epilepsy.

**Methods:** A systematic search was performed on MEDLINE, Embase, Cochrane Library, CBM, CNKI, IDB, VIP Database, and Wanfang Database from inception to 2021 March, and all studies assessing the safety of LCM were included. A meta-analysis was performed for safety data of LCM.

**Results:** Eighty-three studies involving 12268 populations (11 randomized clinical trials (RCTs), 16 cohort studies, 53 case series, and 3 case reports) were included in our study. Meta-analysis of the total incidence of adverse events (AEs) of LCM was 38.7% [95% CI (35.1%, 45.8%); *n*=75 studies]. Incidence of withdrawal due to AEs was 10.8% [95% CI (9.1%, 12.6%); *n*=56 studies], and incidence of serious adverse events (SAEs) was 6.5% [95% CI (4.0%, 8.9%); *n*=13 studies]. Most AEs were in the nervous system and digestive system. The most common AEs were sedation (15.8%), dizziness (15.7%), fatigue (9.4%), and nausea/vomiting (9.3%). For children, the total incidence of AEs of LCM was 32.8% [95% CI (21.6%, 44.0%); *n*=16 studies], and the most common AEs were dizziness (8.6%), nausea/vomiting (8.6%), and somnolence (6.8%).

**Conclusion:** Lacosamide is generally safe and well tolerated in patients with epilepsy. Common AEs were sedation, dizziness, and fatigue. It is necessary to pay more attention to the prevention and management of these AEs and conduct more large-scale and high-quality studies to update safety data.

## Introduction

Epilepsy is one of the most common neurological disorders, affecting over 50 million people worldwide (U.S. National Library of Medicine, 2020). According to a meta-analysis, the prevalence of epilepsy among the general population is 7‰ worldwide, which ranged from 2.3 to 15.9 per 1,000 in developed countries and from 3.6 to 15.4 per 1,000 in developing countries (Aneja and Jain, 2014; Bell et al., 2014). The main treatment for epilepsy is drug therapy. However, approximately one-third of patients do not achieve complete control of seizures with currently available ASMs (Moshe et al., 2015). The increased social and economic burden on patients and health care systems highlight epilepsy as an important public health concern.

As a third generation antiseizure medication (ASM), lacosamide (LCM) enhances the slow inactivation of voltage-gated sodium channels. Classic ASMs such as oxcarbazepine (OXZ), carbamazepine (CBZ), and lamotrigine (LTC) target sodium channels, acting on their fast inactivation; however, LCM has a unique structure and is currently the only highly selective blocker that acts on slow inactivation of sodium channels. LCM has advantage in pharmacokinetic characteristics such as rapid absorption, high oral bioavailability, not affected by food, and low interindividual and intra-individual differences. In contrast to traditional ASMs, newer ASMs decrease drug interactions and enable freedom from seizures (Stephen and Brodie, 2011).

The use of LCM as monotherapy for focal-onset epilepsy has been approved by the Food and Drug Administration (FDA) since September 2014 (Fong et al., 2017). In 2018, it was approved as an add-on treatment for adults and adolescents (age ≥ 16 years) with focal epilepsy in China. The practice guidelines in Belgium (Boon et al., 2021) to date recommend LCM as registered and reimbursed ASMs for add-on treatment for focal-onset seizures in adults and children. Among the newer ASMs, LCM has been increasingly used for acute seizures and status epilepticus in the intensive care unit (Chimakurthy et al., 2020). Because of the efficacy for focal epilepsy (Weston et al., 2015), LCM is clinically widely used in patients with epilepsy. However, there is no conclusive evidence for the safety of LCM in patients with epilepsy, especially in children. A literature review showed that the adverse effects of LCM have gradually emerged, especially some rare adverse events (Li et al., 2020). A multicenter study revealed that the AEs might be enhanced when LCM was used in combination with other sodium channel blockers (Giráldez et al., 2015). The recent systematic review (Strzelczyk et al., 2017) on the safety of LCM only included 9 retrospective cohort studies involving 522 patients with epilepsy. In another systematic review (Zaccara et al., 2013) including only 10 RCTs, no quantitative synthesis of safety data of LCM was conducted. Although both studies stated that LCM was well tolerated, the type and number of included studies were limited, and the sample size of included patients was small. Therefore, it is necessary to update the safety data and conduct a meta-analysis with currently available data for the safety of LCM in patients with epilepsy.

In this study, we conducted a meta-analysis that included RCTs, cohort studies, case series, and case reports to evaluate the safety evidence regarding LCM use in patients with epilepsy.

## Methods

The meta-analysis was conducted according to the Preferred Reporting Items for Systematic Reviews and Meta Analyses (PRISMA) guidelines. Ethical approval and informed consent were not necessary because the purpose of the meta-analysis was to summarize previous studies.

### Search Method

We performed a systematic literature review on MEDLINE, Embase, Cochrane Library, Chinese Biomedical Literature Database, China Knowledge Resource, Integrated Database, VIP Database, and Wanfang Database for literature published from inception to 2021 March. The search strategy was as follows: (“epilepsy” or “epilepsies” or “seizure” or “seizures”) and (“safe” or “safety” or “tolerate” or “tolerability” or “adverse event” or “AE” or “adverse drug reaction” or “ADR”). The search was restricted to human studies, and the language was restricted to English.

### Inclusion and Exclusion Criteria

Trials were included in the meta-analysis if they meet the following criteria. Population: patients with all types of epilepsy and without age limit. Intervention: LCM as monotherapy or add-on therapy. Comparison: for RCTs and cohort studies, the comparison was placebo or other types of pharmacotherapies. For case series, there was no comparison. Outcome: incidence of AEs, incidence of withdrawal due to adverse events, and incidence of SAEs. Study design: all types of studies were included, including RCTs, cohort studies, case series, and case reports.

Trials were excluded if patients were not taking LCM treatment, the safety data of LCM could not be obtained, they were animal experiment or *in vitro* experiment, and they were literature review, systematic review, or meta-analysis.

### Data Collection and Extraction

Two authors independently extracted relevant data and evaluated the methodological quality of the studies. If there was any disagreement, it would be resolved through discussion or consultation by a third evaluator. The data extraction included the following variables and outcomes: study, study type, age, sample, race, country, type of seizure, epilepsy course, diagnostic criteria, intervention of the treatment group, intervention of the control group, follow-up duration, incidence of total adverse event, incidence of withdrawal due to adverse events, and incidence of serious adverse events.

### Quality Assessment

The Cochrane Collaboration’s tool for assessing risk of bias was used to evaluate the quality of RCTs (Higgins et al., 2019). We used the Newcastle–Ottawa Scale (NOS) (Stang, 2010) to evaluate cohort studies. For case series studies, we used the National Institute for Clinical Excellence Guideline checklist to evaluate quality and calculate the mean point of all included studies (NICE, 2002). For case reports, we used the consensus-based clinical case report (CARE) guidelines to evaluate their standardization (Gagnier et al., 2014).

### Statistical Analysis

All statistical analyses were performed using Stata 12.0 (StataCorp, College Station, TX) and Review Manager 5.3. The incidence rates of AEs in LCM were reported with the prevalence and 95% confidence intervals (CI). Significance of evidence was evaluated using the Z-test. We used the *Q* test and *I* ^2^ statistic to assess heterogeneity. If the *Q* test results was *p* < 0.05 and *I* ^2^ > 50%, which represented significant heterogeneity, a random-effects model was applied to evaluate the summary results; in all other cases, we applied a fixed-effects model.

## Results

### Included Studies

**FIGURE 1:**
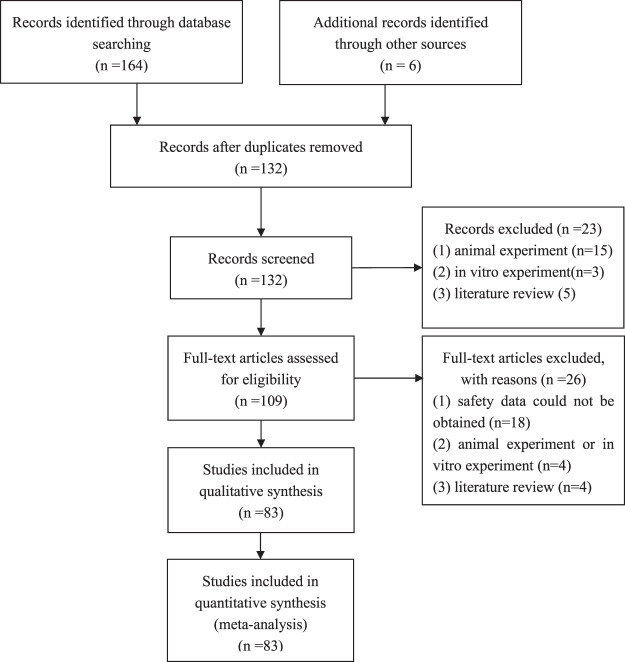
Flowchart of literature search and study inclusion procedure.

After the initial search, we identified 170 studies. Of these, 83 studies met inclusion criteria and were included as the full text. Of the 80 included studies, 11 were RCTs, 16 were cohort studies, 53 were case series, and 3 were case reports. The characteristics of included studies are summarized in Supplementary Table S1 and Figure 1.

The eleven RCTs (Ben-Menachem et al., 2007; Halasz et al., 2009; Chung et al., 2010; Wechsler et al., 2014; Wu et al., 2015; Yang, 2015; Hong et al., 2016; Huang et al., 2018; Schmitz et al., 2020; Vossler et al., 2020; Shin et al., 2021) included 3,364 patients aged from 7 to 70 years, with one study involving 60 children under the age of 18. Most patients were diagnosed with focal-onset seizures. All eleven RCTs were multicentered, and most studies were conducted in the United States (six studies), followed by three studies in China, one in Korea and one in Canada.

Of the sixteen cohort studies (Villanueva et al., 2012; Fountain et al., 2013; Verrotti et al., 2013; Brodie et al., 2014; Giraldez et al., 2015; Zadeh et al., 2015; McGinnis and Kessler, 2016; Kurth et al., 2017; Sarkis et al., 2017; Dogan et al., 2018; Maloney et al., 2018; Neal et al., 2018; Villanueva et al., 2018; Chiara Del et al., 2019; Ferreira et al., 2019; Liu et al., 2020), nine were retrospective cohort studies, six were prospective studies, and one was an ambidirectional cohort study. These studies included totally 2,794 patients aged 1 month to 92 years, with two studies involving a total of 106 children. Most patients were diagnosed with focal-onset seizures. Five studies were conducted in the United States, three in Spain, one in the United Kingdom, one in Belgium, one in Germany, one in Turkey, one in Ireland, one in Australia, and one in Italy and Germany.

The fifty-three case series (Guilhoto et al., 2010; García-Morales et al., 2011; Gavatha et al., 2011; Höfler et al., 2011; Maschio et al., 2011; Casas-Fernández et al., 2012; Heyman et al., 2012; Flores et al., 2012; Harden et al., 2012; Husain et al., 2012; Song et al., 2012; Giorgi et al., 2013; Kamel et al., 2013; Li et al., 2013; Villanueva et al., 2013; Garcés et al., 2014; Grosso et al., 2014; Kellinghaus et al., 2014; Kim et al., 2014; Rosenfeld et al., 2014; Stephen et al., 2014; Wechsler et al., 2014; Yorns et al., 2014; Andrade-Machado et al., 2015; Geffrey et al., 2015; IJff et al., 2015; Lattanzi et al., 2015; Runge et al., 2015; Villanueva et al., 2015; Arkilo et al., 2016; Borzì et al., 2016; Miskin et al., 2016; Vossler et al., 2016; Baulac et al., 2017; Bottcher et al., 2017; Brenner et al., 2017; Rinesalo et al., 2017; Sepúlveda-Sánchez et al., 2017; Svendsen et al., 2017; Arabi et al., 2018; Baker et al., 2018; Munoz-Lopetegi et al., 2018; Ngampoopun et al., 2018; Rosati et al., 2018; Rudà et al., 2018; Samarasekera et al., 2018; Sanmartí-Vilaplana et al., 2018; Toledo et al., 2018; Kleist et al., 2019; Ruegger et al., 2019; Chiara Del et al., 2019; Hmaimess et al., 2020; Ruda et al., 2020; Zhao et al., 2021) included totally 6,107 patients aged 6 months to 95 years, with thirteen studies involving a total of 801 children under the age of 18. Most patients were diagnosed with focal-onset seizures. Eight studies were conducted in Spain, seven in the United States, six in Italy, five in Germany, and four in Australia, two in China, two in the United Kingdom, two in Lebanon, and two in Netherlands.

The three case reports (Stamm, 2020; Krause et al., 2011) included totally three patients aged from 32 to 89 years. One study was conducted in German and one was in France.

### Quality Assessment

For the methodology quality assessment of RCTs, four studies (36.4%) were of low risk in describing using an appropriate method of random sequence generation, and three studies (27.3%) were of low risk in describing using a method to conceal the allocation sequence. We detected performance bias in part of RCTs because only two studies (25.0%) clearly demonstrated blinding of study participants and personnel. For detection bias, three studies (37.5%) described blinding of outcome assessment and two (25.0%) were prone to high risk of bias. Six studies (75.0%) described completeness of outcome data for each main outcome. Reporting bias and other bias were not found in any of the eight studies, as given in Supplementary Table S2.

For the sixteen cohort studies, the mean point estimate was 6.3 (3.2 for selection, 1.4 for comparability, and 1.7 for outcome). Thirteen studies (81.3%) achieved over 5 points, whereas only one study (6.3%) scored 4 points and two studies (12.5%) scored 5 points, which showed that most cohort studies demonstrated good quality.

For the fifty-four case studies, the mean NICE guideline checklist score was 5.4. All case studies clearly described the objective of the study, and 51 studies (96.2%) clearly described the main finding. However, only 12 studies (22.6%) were multicenter studies, and only 6 studies (11.3%) stratified outcomes.

For the three case reports, they all had clear and standard titles, keywords, abstract, introduction, clinical findings, timeline description, treatment, and intervention and discussion. However, only one study (33.3%) reported patient’s view and informed consent.

### Adverse Drug Events

Across all studies, the meta-analysis of total incidence of AEs of LCM was 38.7% [95% CI (35.1%, 45.8%); *n* = 75 studies]. Incidence of withdrawal due to AEs was 10.8% [95% CI (9.1%, 12.6%); *n* = 56 studies], and the incidence of SAEs was 6.5% [95% CI (4.0%, 8.9%); *n* = 13 studies].

For all included studies, the meta-analysis showed that most AEs involved the nervous system and digestive system, followed by the respiratory system and circulatory system. The most common AEs were sedation, dizziness, nasopharyngitis, and fatigue.

For 16 studies which only included children under the age of 18, the total incidence of AEs of LCM was 32.8% [95% CI (21.6%,44.0%); *n* = 16 studies], as given in Table 1 and Table 2. The meta-analysis showed that most AEs involved the nervous system and digestive system. The most common AEs were dizziness [8.6%; 95% CI (4.8%, 12.8%)], nausea/vomiting [8.6%; 95% CI (4.3%, 12.9%)] and somnolence [6.8%; 95% CI (3.7%, 10.0%)].

**TABLE 1:** Meta-analysis of safety outcomes in different systems.

System	Group	Study	*n*/*N*	Heterogeneity	Incidence rate (95% CI)	*P*
Nervous system	Sedation	8	190/1,317	*P* = 0.000, *I* ^2^ = 95.1%	0.158 (0.088, 0.228)	<0.001
—	Dizziness	59	1849/9,334	*P* = 0.000, *I* ^2^ = 95.8%	0.157 (0.127, 0.187)	0.0125
—	Fatigue	21	384/3,462	*P* = 0.000, *I* ^2^ = 88.2%	0.094 (0.067, 0.121)	<0.001
—	Somnolence/drowsiness	41	502/6,421	*P* = 0.000, *I* ^2^ = 91.1	0.079 (0.063, 0.094)	<0.001
—	Headache	37	613/7,258	*P* = 0.000, *I* ^2^ = 89.8%	0.066 (0.051, 0.082)	<0.001
—	Vertigo	6	77/1,405	*P* = 0.000, *I* ^2^ = 89.1%	0.060 (0.025, 0.095)	0.001
—	Diplopia/double version	28	404/5,521	*P* = 0.000, *I* ^2^ = 86.6%	0.057 (0.042, 0.072)	<0.001
—	Ataxia	17	201/2,978	*P* = 0.000, *I* ^2^ = 83.7%	0.056 (0.037, 0.074)	<0.001
—	Vision blurred	10	145/2,276	*P* = 0.000, *I* ^2^ = 85.6%	0.051 (0.028, 0.074)	<0.001
—	Nystagmus	6	85/1,331	*P* = 0.000, *I* ^2^ = 91.4%	0.050 (0.016, 0.084)	0.004
—	Gait disturbance	6	68/1,467	*P* = 0.000, *I* ^2^ = 81.9%	0.045 (0.020, 0.069)	<0.001
—	Anxiety	9	60/1,368	*P* = 0.021, *I* ^2^ = 55.8%	0.040 (0.023, 0.057)	<0.001
—	Depression	9	72/1952	*P* = 0.000, *I* ^2^ = 85.5%	0.033 (0.014, 0.051)	<0.001
—	Insomnia	7	33/874	*P* = 0.009, *I* ^2^ = 64.9%	0.027 (0.007, 0.046)	0.007
—	Memory impairment	3	6/309	*P* = 0.913, *I* ^2^ = 0.0%	0.019 (0.004, 0.034)	0.015
—	Asthenia	3	6/248	*P* = 0.496, *I* ^2^ = 0.0%	0.019 (0.002, 0.036)	0.028
—	Paresthesia/cognitive side effect	5	15/1,052	*P* = 0.053, *I* ^2^ = 57.3%	0.011 (0.000, 0.021)	0.047
Digestive system	Nausea/vomiting	41	751/6,540	*P* = 0.000, *I* ^2^ = 93.1%	0.093 (0.072, 0.115)	<0.001
—	Dyspepsia	1	4/59	Not applicable	0.068 (0.004, 0.132)	0.038
—	Gastrointestinal distress/diarrhoea	12	93/2,204	*P* = 0.000, *I* ^2^ = 76.8%	0.036 (0.021, 0.051)	<0.001
Respiratory system	Nasopharyngitis	7	231/1948	*P* = 0.000, *I* ^2^ = 86.2%	0.115 (0.077, 0.154)	<0.001
—	Upper respiratory tract infection	5	160/1,458	*P* = 0.000, *I* ^2^ = 87.9%	0.097 (0.055, 0.140)	<0.001
—	Influenza	1	9/456	Not applicable	0.020 (0.007, 0.033)	0.002
Circulatory system	Atrioventricular block	1	1/98	Not applicable	0.059 (0.008, 0.442)	0.006
—	ECG PR interval prolongation	1	1/98	Not applicable	0.059 (0.008, 0.442)	0.006
—	Bradycardia	2	2/169	*P* = 0.58, *I* ^2^ = 72.2%	0.025 (0.002, 0.369)	0.007
—	Palpitation	3	7/500	*P* = 1.000, *I* ^2^ = 0.0%	0.014 (0.004, 0.024)	0.008
Locomotor system	Falls	3	54/498	*P* = 0.000, *I* ^2^ = 87.4%	0.055 (0.011, 0.265)	0.097
—	Tremor	19	180/4,045	*P* = 0.000, *I* ^2^ = 75.9%	0.038 (0.025, 0.051)	<0.001
—	Behavior disorders	5	32/708	*P* = 0.000, *I* ^2^ = 83.9%	0.030 (0.006, 0.054)	0.013
Skin system	Irritability	13	54/1,601	*P* = 0.001, *I* ^2^ = 65.2%	0.027 (0.013, 0.041)	<0.001
—	Rash	20	74/3,158	*P* = 0.566, *I* ^2^ = 0.0%	0.018 (0.014, 0.023)	<0.001
—	Pruritus	1	1/66	Not applicable	0.015 (0.002, 0.111)	0.314
Others	Dry mouth	1	6/100	Not applicable	0.060 (0.013, 0.107)	0.012
—	Back pain	1	2/64	Not applicable	0.032 (0.008, 0.123)	0.151
—	Chest pain	2	5/170	*P* = 0.957, *I* ^2^ = 0.0%	0.029 (0.004, 0.055)	0.023
—	Weight gain	1	7/322	Not applicable	0.022 (0.006, 0.038)	0.007
—	Abdominal pain	4	8/293	*P* = 0.070, *I* ^2^ = 57.5%	0.027 (0.008, 0.095)	<0.001
—	Weight loss	5	6/888	*P* = 0.040, *I* ^2^ = 60.0%	0.012 (0.003, 0.046)	<0.001
Any adverse event	—	75	4,581/9,839	*P* = 0.000, *I* ^2^ = 98.8%	0.387 (0.315, 0.458)	<0.001
Any adverse event (studies only including children)	—	17	355/1,051	*P* = 0.000, *I* ^2^ = 93.3%	0.349 (0.244, 0.454)	<0.001

**TABLE 2:** Meta-analysis of safety outcomes of lacosamide vs. placebo or other ASMs.

	Group	Study type	Studies	*n*/*N*1	*n*/*N*2	Heterogeneity	RR (95%CI)	P
Nervous system								
Dizziness	LCM vs. TPM	Cohort study	1	5/160	0/135	Not applicable	9.29 (0.52, 166.53)	0.13
—	LCM vs. ZNS	Cohort study	2	12/179	0/152	*P* = 0.97; *I* ^2^ = 0%	9.32 (1.26, 68.84)	0.03
—	LCM vs. LEV	Cohort study	2	7/182	3/160	*P* = 0.11; *I* ^2^ = 61%	2.10 (0.58, 7.54)	0.26
—	LCM vs. PGB	Cohort study	1	5/160	0/135	Not applicable	9.29 (0.52, 166.53)	0.13
—	LCM vs. PER	Cohort study	1	16/70	11/70	Not applicable	1.45 (0.73, 2.91)	0.29
—	LCM vs. CBZ	RCT	1	8/64	10/62	Not applicable	0.74 (0.27, 2.03)	0.56
—	LCM vs. placebo	RCT	4	383/1,307	46/548	*P* = 0.68; *I* ^2^ = 0%	3.28 (2.45, 4.38)	<0.001
—	—	Cohort study	1	37/385	5/179	Not applicable	3.70 (1.48, 9.25)	0.005
Sedation	LCM vs. TPM	Cohort study	1	5/160	0/135	Not applicable	9.29 (0.52, 166.53)	0.13
—	LCM vs. ZNS	Cohort study	1	5/160	14/141	Not applicable	0.31 (0.12, 0.85)	0.02
—	LCM vs. LEV	Cohort study	1	5/160	20/136	Not applicable	0.21 (0.08, 0.55)	0.001
—	LCM vs. PGB	Cohort study	1	5/160	18/135	Not applicable	0.23 (0.09, 0.61)	0.003
Somnolence/drowsiness	LCM vs. PER	Cohort study	1	6/70	29/70	Not applicable	0.21 (0.09, 0.47)	<0.001
—	LCM vs. CBZ	RCT	1	1/64	8/62	Not applicable	0.11 (0.01, 0.99)	0.04
—	LCM vs. placebo	RCT	3	95/985	20/385	*P* = 0.44; *I* ^2^ = 0%	1.79 (1.12, 2.86)	0.01
—	—	Cohort study	1	10/358	2/179	Not applicable	2.50 (0.55, 11.29)	0.23
Headache	LCM vs. TPM	Cohort study	1	4/160	7/135	Not applicable	0.48 (0.14, 1.61)	0.24
—	LCM vs. ZNS	Cohort study	1	4/160	6/141	Not applicable	0.59 (0.17, 2.04)	0.40
—	LCM vs. LEV	Cohort study	1	4/160	4/136	Not applicable	0.85 (0.22, 3.33)	0.82
—	LCM vs. PGB	Cohort study	1	4/160	0/135	Not applicable	7.60 (0.41, 139.95)	0.17
—	LCM vs. CBZ	RCT	1	8/64	9/62	Not applicable	0.84 (0.30, 2.34)	0.74
—	LCM vs. placebo	RCT	4	164/1,307	44/548	*P* = 0.65; *I* ^2^ = 0%	1.49 (1.08, 2.04)	0.01
Diplopia/double version	LCM vs. TPM	Cohort study	1	3/160	3/135	Not applicable	0.84 (0.17, 4.11)	0.83
—	LCM vs. ZNS	Cohort study	1	3/160	0/141	Not applicable	6.17 (0.32, 118.50)	0.23
—	LCM vs. LEV	Cohort study	1	3/160	0/136	Not applicable	5.96 (0.31, 114.31)	0.24
—	LCM vs. PGB	Cohort study	1	3/160	5/135	Not applicable	0.51 (0.12, 2.08)	0.35
—	LCM vs. placebo	RCT	2	60/643	4/260	*P* = 0.66; *I* ^2^ = 0%	5.86 (2.15, 15.94)	<0.001
—	—	Cohort study	1	7/358	1/179	Not applicable	3.50 (0.43, 28.23)	0.24
Ataxia/balance disorder	LCM vs. TPM	Cohort study	1	4/160	0/135	Not applicable	7.60 (0.41, 139.95)	0.17
—	LCM vs. ZNS	Cohort study	1	4/160	3/141	Not applicable	1.18 (0.27, 5.16)	0.83
—	LCM vs. LEV	Cohort study	1	4/160	0/136	Not applicable	7.66 (0.42, 140.98)	0.17
—	LCM vs. PGB	Cohort study	1	4/160	9/135	Not applicable	0.38 (0.12, 1.19)	0.10
—	LCM vs. PER	Cohort study	1	3/70	4/70	Not applicable	0.75 (0.17, 3.23)	0.70
—	LCM vs. placebo	RCT	3	88/944	6/364	*P* = 0.83; *I* ^2^ = 0%	5.03 (2.23, 11.37)	<0.001
—		Cohort study	1	6/358	1/179	Not applicable	3.00 (0.36, 24.73)	0.31
Nystagmus	LCM vs. placebo	RCT	2	45/622	10/201	*P* = 0.51; *I* ^2^ = 0%	1.45 (0.74, 2.82)	0.28
Paresthesia/cognitive side effect	LCM vs. TPM	Cohort study	1	0/160	8/135	Not applicable	0.05 (0.00, 0.85)	0.04
—	LCM vs. ZNS	Cohort study	2	6/179	4/152	Not applicable	1.27 (0.37, 4.43)	0.70
Fatigue	LCM vs. TPM	Cohort study	1	0/160	17/135	Not applicable	0.02 (0.00, 0.40)	0.009
—	LCM vs. ZNS	Cohort study	2	4/179	2/152	Not applicable	1.70 (0.32, 9.14)	0.54
—	LCM vs. LEV	Cohort study	1	0/160	4/136	Not applicable	0.09 (0.01, 1.74)	0.11
—	LCM vs. PGB	Cohort study	1	0/160	6/135	Not applicable	0.06 (0.00, 1.14)	0.06
—	LCM vs. PER	Cohort study	1	0/70	4/70	Not applicable	0.11 (0.01, 2.03)	0.14
—	LCM vs. CBZ	RCT	1	5/64	9/62	Not applicable	0.50 (0.16, 1.58)	0.24
—	LCM vs. placebo	RCT	2	63/643	11/260	*P* = 0.37; *I* ^2^ = 0%	2.04 (1.08, 3.85)	0.03
Vertigo	LCM vs. PGB	Cohort study	1	0/160	6/135	Not applicable	0.06 (0.00, 1.14)	0.06
—	LCM vs. placebo	RCT	1	21/322	3/163	Not applicable	3.54 (1.07, 11.71)	0.04
—	—	Cohort study	1	3/158	0/179	Not applicable	3.51 (0.18, 67.58)	0.41
Anxiety	LCM vs. TPM	Cohort study	1	0/160	7/135	Not applicable	0.06 (0.00, 0.98)	0.05
—	LCM vs. ZNS	Cohort study	1	0/160	7/141	Not applicable	0.06 (0.00, 1.02)	0.05
—	LCM vs. PGB	Cohort study	1	0/160	4/135	Not applicable	0.09 (0.01, 1.73)	0.11
—	LCM vs. CBZ	RCT	1	2/64	4/62	Not applicable	0.47 (0.08, 2.65)	0.39
Vision blurred	LCM vs. placebo	RCT	2	75/622	8/201	*P* = 0.37; *I* ^2^ = 0%	2.89 (1.42, 5.89)	0.004
—	—	Cohort study	1	8/358	0/179	Not applicable	8.52 (0.49, 146.85)	0.14
Insomnia	LCM vs. CBZ	RCT	1	1/64	2/62	Not applicable	0.48 (0.04, 5.39)	0.55
Depression	LCM vs. CBZ	RCT	1	3/64	0/62	Not applicable	7.11 (0.36, 140.62)	0.20
Digestive system								
Nausea/vomiting	LCM vs. TPM	Cohort study	1	6/160	0/135	Not applicable	10.98 (0.62, 193.17)	0.10
—	LCM vs. ZNS	Cohort study	1	6/160	13/141	Not applicable	0.41 (0.16, 1.04)	0.06
—	LCM vs. LEV	Cohort study	1	6/160	0/136	Not applicable	11.06 (0.63, 194.60)	0.10
—	LCM vs. PGB	Cohort study	1	6/160	3/135	Not applicable	1.69 (0.43, 6.62)	0.45
—	LCM vs. PER	Cohort study	1	3/70	0/70	Not applicable	7.00 (0.37, 133.06)	0.20
—	LCM vs. CBZ	RCT	1	10/64	10/62	Not applicable	0.96 (0.37, 2.50)	0.94
—	LCM vs. placebo	RCT	3	203/944	25/364	*P* = 0.33; *I* ^2^ = 10%	2.82 (1.85, 4.28)	<0.001
—	—	Cohort study	1	5/358	0/179	Not applicable	5.52 (0.31, 99.19)	0.25
Abdominal pain	LCM vs. ZNS	Cohort study	1	0/160	4/141	Not applicable	0.10 (0.01, 1.80)	0.12
Circulatory system								
Palpitation	LCM vs. placebo	Cohort study	1	5/358	0/179	Not applicable	5.52 (0.31, 99.19)	0.25
Respiratory system								
Nasopharyngitis	LCM vs. CBZ	RCT	1	8/64	6/62	Not applicable	1.33 (0.43, 4.09)	0.62
—	LCM vs. placebo	RCT	2	70/685	29/347	*P* = 0.59; *I* ^2^ = 0%	1.21 (0.81, 1.83)	0.35
Upper respiratory tract infection	LCM vs. placebo	RCT	2	56/684	22/281	*P* = 0.53; *I* ^2^ = 0%	0.98 (0.61, 1.57)	0.94
Locomotor system								
Tremor	LCM vs. TPM	Cohort study	1	4/160	3/135	Not applicable	1.13 (0.26, 4.94)	0.88
—	LCM vs. ZNS	Cohort study	1	4/160	0/141	Not applicable	7.94 (0.43, 146.15)	0.16
—	LCM vs. LEV	Cohort study	1	4/160	0/136	Not applicable	7.66 (0.42, 140.98)	0.17
—	LCM vs. PGB	Cohort study	1	4/160	0/135	Not applicable	7.60 (0.41, 139.95)	0.17
—	LCM vs. CBZ	RCT	1	2/64	6/62	Not applicable	0.30 (0.06, 1.55)	0.15
—	LCM vs. placebo	RCT	1	33/301	8/104	Not applicable	1.43 (0.68, 2.99)	0.35
Falls	LCM vs. PER	Cohort study	1	1/70	1/70	Not applicable	1.00 (0.06, 15.67)	1
Skin system								
Irritability	LCM vs. TPM	Cohort study	1	0/160	9/135	Not applicable	0.04 (0.00, 0.76)	0.03
—	LCM vs. PER	Cohort study	1	0/70	3/70	Not applicable	0.14 (0.01, 2.72)	0.20
Rash	LCM vs. TPM	Cohort study	1	4/160	0/135	Not applicable	7.60 (0.41, 139.95)	0.17
—	LCM vs. ZNS	Cohort study	1	4/160	6/141	Not applicable	0.59 (0.17, 2.04)	0.40
—	LCM vs. LEV	Cohort study	1	4/160	0/136	Not applicable	7.66 (0.42, 140.98)	0.17
—	LCM vs. PGB	Cohort study	1	4/160	0/135	Not applicable	7.60 (0.41, 139.95)	0.17
Other systems								
Weight loss	LCM vs. TPM	Cohort study	1	0/160	12/135	Not applicable	0.03 (0.00, 0.57)	0.02
—	LCM vs. ZNS	Cohort study	1	0/160	6/141	Not applicable	0.07 (0.00, 1.19)	0.07
Any AEs	LCM vs. TPM	Cohort study	1	36/160	42/135	Not applicable	0.72 (0.49, 1.06)	0.10
—	LCM vs. ZNS	Cohort study	2	55/231	70/180	*P* = 0.12; *I* ^2^ = 58%	0.67 (0.39, 1.15)	0.15
—	LCM vs. LEV	Cohort study	2	38/182	49/160	*P* = 0.92; *I* ^2^ = 0%	0.67 (0.46, 0.96)	0.03
—	LCM vs. PGB	Cohort study	1	36/160	68/135	Not applicable	0.45 (0.32, 0.62)	<0.001
—	LCM vs. PER	Cohort study	1	32/70	51/70	Not applicable	0.63 (0.47, 0.84)	0.002
—	LCM vs. CBZ	RCT	1	52/64	56/62	Not applicable	0.46 (0.16, 1.33)	0.15
—	LCM vs. placebo	RCT	2	532/684	196/281	*P* = 0.11; *I* ^2^ = 60%	1.11 (0.96, 1.28)	0.15
—	—	Cohort study	1	111/358	35/179	Not applicable	1.59 (1.13, 2.22)	0.007

ASMs, antiseizure medications; LCM, lacosamide; TPM, topiramate; ZNS, zonisamide; LEV, levetiracetam; PGB, pregabalin; PER, perampanel; CBZ, carbamazepine; RCT, randomized clinical trial; *N*1, lacosamide group; *N*2, control group.

#### Nervous System

In total, seventeen types of adverse drug events involving the nervous system were reported. Meta-analysis of 8 studies showed that sedation was the most common AE with the highest incidence [15.8%, 95% CI (8.8%, 22.8%)]. Dizziness was the next most common AE with the incidence of 15.7% [95% CI (12.7%, 18.7%)] and was frequently reported in 59 studies. The following were fatigue [9.4%; 95% CI (6.7%, 12.1%)], somnolence [7.9%; 95% CI (6.3%, 9.4%)], and headache [6.6%; 95% CI (5.1%, 8.2%)]. Furthermore, ataxia and blurred vision were also frequently reported.

#### Digestive System

In total, four types of AEs involving the digestive system were reported. Nausea/vomiting was the most common AE involving the digestive system, which had an incidence of 9.4% [95% CI (7.0%, 11.7%)]. This was followed by dyspepsia, which had an incidence of 6.8% [95% CI (0.4%, 13.2%)].

#### Respiratory System

Three types of AEs were reported across these studies: nasopharyngitis [11.5%; 95% CI (7.7%, 15.4%)], upper respiratory tract infection [9.7%; 95% CI (5.5%, 14.0%)], and influenza [2.0%; 95% CI (0.7%, 3.3%)]. Incidence of AEs involving the respiratory system was relatively high, but these three AEs were only reported in 7 studies.

#### Circulatory System

AEs with the use of LCM in the circulatory system were reported rarely. Both atrioventricular block and ECG PR interval prolongation were reported in only one studies, both with an incidence of 5.9% [95% CI (0.8%, 44.2%)]. Bradycardia was reported in two studies, which had an incidence of 2.5% [95% CI (0.2%, 36.9%)].

#### Locomotor System

Falls was the most common AE in the locomotor system, which was reported in 3 studies with the incidence of 5.5% [95% CI (1.1%, 26.5%)]. The next most common AEs in the locomotor system was tremor [3.8%; 95% CI (2.5%, 5.1%)], followed by behavior disorders [3.0%; 95% CI (0.6%, 5.4%)].

#### Skin System

Figure 1 shows that irritability and rash were commonly reported in patients treated with LCM, which had incidences of 2.7% [95% CI (1.3%, 4.1%)] and 1.8% [95% CI (1.4%, 2.3%)], respectively.

#### Others

Other AEs were less common, such as weight loss, weight gain, dry mouth, and chest pain. Weight loss was reported in five studies and had an incidence of 1.2% [95% CI (0.3%, 4.6%)].

### Lacosamide Versus Placebo or Other Antiseizure Medications

#### Lacosamide Versus Placebo

We retrieved four RCTs (Ben-Menachem et al., 2007; Halasz et al., 2009; Chung et al., 2010; Hong et al., 2016) that compared 13 types of AEs between LCM and placebo use. Overall, the total incidence of AEs between the two groups was not of significantly different (*p* = 0.15). However, there were significant differences between groups for all AEs involving the nervous system (dizziness, sedation, headache, diplopia, fatigue, vertigo, and blurred vision; *p* < 0.05). As for nausea/vomiting, the incidence in patients with the use of LCM was significantly higher than that in the placebo group [RR = 2.82; 95% CI (1.85, 4.28); *p* <.001]. No significant difference was found between groups in the incidence of AEs involving other systems, such as tremor, nystagmus, nasopharyngitis, and upper respiratory tract infection (*p* > 0.05).

#### Lacosamide Versus Topiramate

One cohort study (Brodie et al., 2014) focused on AEs with the use of LCM and TPM. Overall, the total incidence of AEs between the two groups was not significantly different (*p* = 0.10). There were significant differences in the incidences of paresthesia [RR = 0.05; 95% CI (0.00, 0.85); *p* = 0.04], fatigue [RR = 0.02; 95% CI (0.00, 0.40); *p* = 0.009], irritability [RR = 0.04; 95% CI (0.00, 0.76); *p* = 0.03], and weight loss [RR = 0.03; 95% CI (0.00, 0.57); *p* = 0.02]. For these four AEs, the incidence in the LCM group was significantly lower than that in the topiramate group. There were no significant differences in incidence of AEs involving other systems, such as the digestive, respiratory, or locomotor systems (*p* > 0.05).

#### Lacosamide Versus Zonisamide

We identified two cohort studies (Brodie et al., 2014; Sarkis et al., 2017) that compared the incidence of AEs between LCM and ZNS use. Total incidence of AEs was not significantly different between the two groups [RR = 0.67; 95% CI (0.39, 1.15); *p* = 0.15]. Of the 13 types of AEs reported across studies, only two involving the nervous system were significantly different between groups, which were dizziness [RR = 9.32; 95% CI (1.26, 68.84); *p* = 0.03] and sedation [RR = 0.31; 95% CI (0.12, 0.85); *p* = 0.02]. As for dizziness, the incidence in the LCM group was significantly higher than that in the zonisamide group.

#### Lacosamide Versus Levetiracetam

Two cohort studies (Brodie et al., 2014; Chiara Del et al., 2019) were identified that compared eight types of AEs with LCM and LEV use. Total incidence of AEs between the two groups was significantly different [RR = 0.67; 95% CI (0.46, 0.96); *p* = 0.03]. For AEs involving the nervous system, there were significant differences in the incidence of sedation between the two drugs [RR = 0.21; 95% CI (0.08, 0.55); *p* = 0.001]. However, no significant differences were found for AEs involving other systems (*p* > 0.05).

#### Lacosamide Versus Pregabalin

Only one cohort study (Chiara Del et al., 2019) reviewed safety outcomes of LCM and PGB use. Total incidence of AEs was significantly different between the two groups [RR = 0.45; 95% CI (0.32, 0.62); R < 0.001]. Incidence of sedation with LCM use was 3.1%, whereas with PGB use was 13.3%, which was significantly different [RR = 0.23; 95% CI (0.09, 0.61); *p* = 0.003]. There were no significant differences in any other AEs.

#### Lacosamide Versus Perampanel

We identified one study (Kurth et al., 2017) that focused on safety outcomes of LCM and PER use. Total incidence of AEs was significantly different and of the LCM groups was lower [RR = 0.63; 95% CI (0.47, 0.84); *p* = 0.002]. No significant differences were found in incidence of any AEs in all systems.

#### Lacosamide Versus Carbamazepine

Meta-analysis of 1 RCT that included 700 patients showed that there was no significant difference in the incidence of AEs between LCM groups and CBZ groups [RR = 0.46; 95% CI (0.16, 13.3); R = 0.15]. A significant difference was observed in the incidence of somnolence between LCM and CBZ treatment groups (RR = 0.11; 95% CI: 0.01, 0.00; *p* = 0.04), in which the incidence in LCM groups was significantly lower.

## Discussion

### Statement of Main Findings

We conducted a systematic review to evaluate the safety and tolerability of LCM in patients with epilepsy, including a total of 83 studies involving 12,268 participants. Results showed that the incidence of AEs with LCM use was 34.9%. Incidence of withdrawal due to AEs was 10.8%, and incidence of SAEs was 6.5%. The most common AE was in the nervous system, next the digestive system. The most common AEs were sedation, dizziness, and fatigue. For studies which only included children under age of 18, the total incidence of AEs of LCM was 32.8% and the most common AEs were dizziness, nausea/vomiting, and somnolence.

### Comparison With Other Studies

Compared with previous publication, our systematic review provided more reliable findings which were based on larger sample size studies with more types of study designs. Therefore, the results might be more convincing.

The results differed from other studies in some respects. BITON’s meta-analysis (Biton et al., 2015) reported that the most common AEs were headache (30.6%), nausea (11.4%), and diplopia (10.5%). In addition, Hong’s study (Hong et al., 2016) showed different results that the most common AEs were in the nervous system (39.0%) and infection (25.3%). Strzelczyk conducted a systematic review (Strzelczyk et al., 2017) and included 9 RCTs showing that the most common AEs were dizziness, abnormal vision, diplopia, nystagmus, and fatigue. Paquette conducted a systematic review (Paquette et al., 2015) and included 27 studies; they found dizziness (21.8%), vision disturbances (10.4%), drowsiness (7.4%), and headache (7.0%) were the most common AEs. These data had something in common with our study results that the most common AEs involved the nervous system, followed by those involving the digestive system. And dizziness and headache were frequently appeared in patients with the use of LCM. However, some results above somehow differed from our study. In our study, sedation was with the highest incidence (15.8%), followed by dizziness (15.7%), which disagreed with the conclusion by Li et al. (2020) that dizziness is the most common adverse events to LCM in all clinical studies. This was because only 8 studies reported the data of sedation, while 59 studies reported dizziness, so the number of included studies had an influence on the result. Besides, for previous studies like the systematic review (Strzelczyk et al., 2017) which only included 9 RCTs, the results were not completely persuasive because the total number of included studies was limited.

Due to the limited number of RCTs that directly compared the safety of different ASMs for epilepsy in included studies, we could not conduct a network meta-analysis, but we compared our results with recent two network meta-analyses of RCTs (Lattanzi et al., 2019a; Lattanzi et al., 2019b). They found that there were no statistically differences in AEs occurrence between CBZ and LCM in monotherapy, and the drug withdrawal rate due to AEs in the LCM treatment group was significantly lower than that in the CBZ group. The results were in accordance with ours. In spite of that, we also found that the incidence of somnolence in LCM treatment groups was significantly lower than that in CBZ treatment groups. So, our results all supported that LCM showed a better tolerability profile than CBZ, leading to lower withdrawal rates.

### Safety Outcome Compared to Other Antiseizure Medications

Over the last 20 years, 13 ASMs have been licensed for adjunctive use, which have mainly been for patients with uncontrolled focal and/or generalized tonic-clonic seizures (Rheims et al., 2011; Brodie, 2013). In the nervous system, the incidence of AEs in the LCM groups was significantly higher than that in the placebo groups. For AEs involving the digestive system, the incidence of nausea/vomiting in LCM groups was significantly higher than that in placebo groups, and this was not observed with other ASMs. For AEs involving the locomotor system, there were no significant differences between LCM and other ASMs. For AEs involving the respiratory system, there were no significant differences between LCM and other ASMs. For AEs involving the skin system, irritability was less frequent with LCM use compared with that with topiramate.

### Limitation and Future Research

Our study also has some limitations. First, we have performed a comprehensive retrieval from inception to 2021 March; it is still possible that unpublished reports were not included. Second, our study focused on patients of all ages and with all kinds of epilepsy, so the safety might differ greatly from children to adults and due to different LCM dosages. Third, the measures and definition of SAEs might differ among the included studies, especially for serious AEs, which might cause clinical heterogeneity.

As for future research, the Common Terminology Criteria for Adverse Events (CTCAE) was suggested as a tool for monitoring adverse events from ASMs (Gay et al., 2011), since we found that there were many studies that still used ambiguous and nonstandard terminology like “worsening seizures.” In addition, some evidences showed that AEs were associated with the dose of LCM and their incidence increased with increasing dose. In the future, further studies should address the issue. Moreover, up to now, LCM is only approved by FDA and CFDA for adults and children of ≥ 4 ages, so more conclusive evidence is needed to testify the safety and tolerability of LCM in children with epilepsy. In our study, the incidence of AEs in children groups is lower than that in patients of all age groups. This was probably because that the number of included study of children was limited, eventhough previous studies have suggested a good level of efficacy and safety for LCM throughout pregnancy and breastfeeding and argue against teratogenic or toxic potentialities (Lattanzi et al., 2017; Maria et al., 2019); however, since LCM appears to cross the near-term placenta freely and could accumulate in a breast-fed infant due to lower renal excretion, more studies are needed to identify the safety of LCM in pregnancy and newborn (Svendsen et al., 2017; Kohn et al., 2020; Landmark et al., 2021).

## Conclusion

Lacosamide is generally safe and well tolerated in patients with epilepsy. Common AEs were sedation, dizziness, and fatigue. It is necessary to pay more attention to the prevention and management of these AEs and conduct more large-scale and high-quality studies to update safety data.

## Data Availability

The original contributions presented in the study are included in the article/Supplementary Material, and further inquiries can be directed to the corresponding authors.
