# Supplementary material for: Safety and Tolerability of Lacosamide in Patients With Epilepsy: A Systematic Review and Meta-Analysis
Source: Front Pharmacol. 2021 Sep 20;12:694381. doi: 10.3389/fphar.2021.694381 (PMC8488108; doi:10.3389/fphar.2021.694381)
Supplement: Supplementary file 1 [file DataSheet1.pdf]

Supplementary table 1: General characteristics of included studies

study	study type	Characteristics of participants							Interventions			Basic safety outcome		
		Age(Mean±SD) (year)	Sample (male)	Race	Country	Type of seizure	Epilepsy course(y)	Diagnostic criteria	Treatment group	Control group	Follow up duration (week)	Incidence of treatment-emergent adverse event	Incidence of withdrawal due to adverse reactions	Incidence of serious adverse events
Shin 2020	RCT	43(31-55)<br>T:38.5(30-50)<br>C1:51(35-57.5)<br>C2:41.5(30.5-54)	70(46)<br>T:22(16)<br>C1:24(15)<br>C2:24(15)	NR	Koera	Focal-onset seizures	13(4-25)<br>T:11(5-21)<br>C1:12(2.75-38)<br>C2:15(5.5-23)	NR	LCM(initial dose:50mg/d, maximum dose:400mg/d; increased weekly by 100mg)	C1:LCM(initial dose:200mg/d, maximum dose:400mg/d; increased to maximum dose at day 8)<br>C2:LCM(initial dose:100mg/d, maximum dose:400mg/d; increased every two days by 100mg)	0.3	46%<br>T:50%<br>C1:42%<br>C2:46%	44.3%	NR
Vossler 2020	RCT	T:27.6±12.5<br>C:27.8±13.1	242<br>T:121(45)<br>C:121(55)	NR	North America; Latin America; Europe; the Asia-Pacific region	Primary generalised tonic-clonic seizures	T:15.4±13.0<br>C:15.5±13.1	ILAE, 2017	LCM(initial dose:2mg/kg/d or 100mg/d; maximum dose:(8–12mg/kg/day for paediatric patients weighing <30kg; 6–8mg/kg/day for paediatric patients weighing ≥30kg to <50kg; 300–400mg/day for adults and paediatric patients weighing ≥50kg; gradually increased weekly)	Placebo	6	T:46.3%<br>C:34.7%	T:9.1%<br>C:5.1%	T:6.6%<br>C:3.3%
Schmitz 2019	RCT	48.0±16.8<br>T:47.6±17.3<br>C:48.4±16.4	126(54)<br>T:64(30)<br>C:62(24)	NR	Europe; North America; the Asia Pacific	Simple focal:42(33.3%); Complex partial:58(46.0%); Partial evolving to secondarily generalized:57(45.2%); Myoclonic:1(0.8%); Tonic-clonic:14(11.1	26.0(16.0-45.0)<br>T:25.0(16.5-38.5)<br>C:28.0(16.0-52.0)	ILAE	LCM(initial dose:100mg/d; maximum dose:400-600mg/d; gradually increase 100mg/d a week)	CBZ-CR(initial dose:200mg/d; maximum dose:800-1200mg/d; gradually increase 200mg/d a week)	6	T:45.3%<br>C:61.3%	T:10.9%<br>C:24.2%	T:12.5%<br>C:3%

						%)								
Huang 2018	RCT	40.8 ±12.9(18-65)<br>T1:40.2± 14.4<br>T2:43.9 ± 12.4<br>T3:38.4 ± 11.6	71<br>T1:23<br>T2:24<br>T3:24	NR	Canada	Refractory Focal Epilepsy	21.7 ± 16.0<br>T1:24.9 ± 18.2<br>T2:20.2 ± 15.8<br>T3:20.2 ±14.1	NR	T1:LCM(100mg,iv>30 min)<br>T2:LCM(200mg,iv>30 min)<br>T3:LCM(400mg,iv>30 min)	/	/	T1:8.7%<br>T2:12.5%<br>T3:29.1%	0	0
Hong 2016	RCT	32.3±11.9(16-70)<br>T1:33.2±12.2(16-70)<br>T2:32.3±11.9(16-70)<br>C:31.8±12.0(16-70)	547(300)<br>T1:183(94)<br>T2:181(104)<br>C:184(102)	Asian	China; Japan	Simple partial:185(34.0%)<br>Complex partial impaired consciousness at onset:367(67.5%)<br>Complex partial with simple partial onset:158(29.0%)<br>Secondarily generalized:350(64.3%)	17.7±11.4<br>T1:18.3±10.9<br>T2:17.9±11.7<br>C:16.8±11.5	ILAE	T1:LCM(initial dose:100mg/d; maximun dose:200mg/d; gradually increased)<br>T2:LCM(initial dose:100mg/d; maximum dose:400mg/d; gradually increased)	Placebo	4	T1:65.0%<br>T2:79.4%<br>C:69.6%	T1:4.4%<br>T2:15.6%<br>C:7.6%	T1:1.1%<br>T2:5.0%<br>C:1.6%
Wu 2015	RCT	T1:29.61±12.74(12.7-69.8)<br>T2:30.50±11.18(13.2-65.7)<br>C:30.54±12.04(14.6-65.8)	515<br>T1:172(97)<br>T2:174(93)<br>C:169(90)	NR	China	T1:Single partial:36(20.93%); Complex partial:102(59.3%); Generalized:8(4.65%)<br>T2:Single partial:38(21.84%); Complex partial:104(59.77%); Generalized:5(2.87%)<br>C:Single partial:33(19.53%):Complex partial:105(62.13%); Generalized:7(4.14%)	T1:13.30±8.43<br>T2:14.42±10.06<br>C:13.23±9.21	ILAE	T1:LCM(initial dose:0mg; maximum dose:200mg/d; gradually increased)<br>T2:LCM(initial dose:100mg/d; maximum dose:400mg/d; gradually increased)	Placebo	4.5	T1:25.84%<br>T2:36.11%<br>C:19.55%	T1:4.47%<br>T2:6.63%<br>T3:3.33%	T1:0<br>T2:1.2%<br>C:1.2%
Yang 2015*	RCT	T:11.12±3.18(7-13)<br>C:11.1±3.21(7-14)	60<br>T:30(15)<br>C:30(17)	Asian	China	T:Partial-onset seizures:8; Complex partial seizures:12; Tonic clonic seizures:8; Tonic seizures:2; Clonic seizures:3<br>C:Partial-onset	T:4.51±3.11<br>C:4.70±3.24	NR	LCM(initial dose:100mg/d; maximum dose:200mg/d; gradually increase 100mg/d a week)	TPM(Initial dose:0.5-1.0mg/kg/d； Maxium dose：4mg/kg/d；gradually increase 0.5-1.0mg/kg/d a week)	16(1.5-48)	T:25.0%<br>C:33.3%	0	0

						seizures:9; Complex partial seizures:10; Tonic clonic seizures:9; Tonic seizures:3; Clonic seizures:2								
Wechsler 2014	RCT	40.6±13.0(16-69) T:41.4±14.3(16-69) C:40.4±12.5(17-69)	425(206) T:319(150) C:106(56)	Black; White; Other	US; other countries	Simple partial:201(47.3%) Complex partial: 363(85.4%) Secondary generalized:287(67.5%)	17.2±14.0 T:16.9 ±13.5 C: 18.0±15.3	ILAE	LCM(initial dose:200mg/d;maxium dose:400mg/d; gradually increased)	LCM(Initial dose:200mg/d;maxium dose:300mg/d; gradually increased)	4.75	84.5% T:85.3% C:84.5%	16.2% T:16.9% C:14.2%	4.0% T:5.3% C:0
Chung 2010	RCT	38.3±12.13(16.0–61.0) T1:39.1±12.37(17.0–71.0) T2:36.8±11.76(16.0–69.0) C:38.1±11.96(16.0–61.0)	405 T1:204(104) T2:97(47) C:104(49)	White; Black; Asian; Other	US	Focal-onset seizures	NR	ILAE	T1:LCM(initial dose:100mg/d; maximum dose:400mg/d; gradually increased) T2:LCM(initial dose:100mg/d; maximum dose:600mg/d; gradually increased)	Placebo	4.5	NR	T1:5% T2:18% C:27%	T1:5.90% T2:3.10% C:2.90%
Halasz 2009	RCT	37.8±11.88(16-70) T1:37.9±12.96(16-66) T2:38.5±10.93(16-70) C:36.9±11.70(17-63)	485 T1:163(90) T2:159(69) C:163(91)	Caucasian; Black; Asian	US	Simple partial-onset seizures:67(41.1%); Complex partial-onset seizures:142(87.1%); Partial-onset seizures with secondary generalization:125(76.7%)	22.3±12.56 T1:22.9 ± 12.30 T2:22.8 ± 13.15 C:21.1 ± 12.23	ILAE, 1981	T1:LCM(initial dose:0mg; maximun dose:200mg/d; gradually increased) T2:LCM(initial dose:100mg/d; maximum dose:400mg/d; gradually increased)	Placebo(100mg/d)	4	NR	8.6% T1:6.1% T2:15.1% C:4.9%	T1:8% T2:9.4% C:3.7%
Ben-Menachem 2007	RCT	T1:39.9±11.71(18-65) T2:41.2±11.61(18-68) T3:39.4±10.53(18-64) C:38.9±11.11(19-66)	418 T1:107(46) T2:108(53) T3:106(45) C:97(47)	Caucasian; Black; Asian; Other	US	Focal-onset seizures	T1:25.1 ± 12.89 T2:24.7 ± 13.08 T3:23.6 ± 12.74 C:24.6 ± 11.77	ILAE	T1:LCM(initial dose:100mg/d; maximun dose:200mg/d; gradually increased) T2:LCM(initial dose:100mg/d; maximum dose:400mg/d; gradually increased) T3:LCM(initial dose:100mg/d; maximum dose:600mg/d; gradually increased)	Placebo(100mg/d)	4.5	T1:79% T2:81% T3:92% C:70%	T1:11.2% T2:18.5% T3:30.2% C:5.2%	T1:9% T2:6% T3:3% C:5%

Liu 2020	Prospective cohort study	T:55.42±4.98<br>C:56.13±5.68	T:120(64)<br>C:132(69)	Asian	China	Refractory partial seizures	T:8.01±3.31<br>C:8.57±3.17	NR	LCM(initial dose:100mg/d, maximum dose:200-400mg/d; increased weekly by 100mg)<br>LEV(initial dose:500mg/d, maximum dose:1000mg/d; increased every two weeks by 500mg)	LEV(initial dose:500mg/d, maximum dose:1000mg/d; increased every two weeks by 500mg)	6	T:25.83%<br>C:20.4%	NR	NR
Del Bianco 2019	Retrospective cohort study	T:76.23±7.5(66-88)<br>C:73.58±6.39(65-88)	T:22(10)<br>C:24(10)	NR	NR	Focal-onset seizures	T:4.23±8.53<br>C:2.29±6.11	NR	LCM(mean dose:204.51±88.51mg/d)	LEV(Mean dose:1281.25±378.15mg/d)	12	T:2/22(9.1%)<br>C:3/24(12.5%)	NR	NR
Ferreira 2019*	Prospective cohort study	7.0±5.1(≥1 month to ≤17)	47(23)	White; Black; Asian; Other	US	Focal-onset seizures	4.3±3.7	ILAE	LCM(initials dose: 2 mg/kg/d; maximum dose:8m-12mg/kg/d; increase the dose by 2 mg/kg/d per week)	/	3.3	87.5%	42.6%	NR
Dogan 2018	Retrospective cohort study	34±14.5 (16-79)	136(70)	NR	Turkey	Focal-onset seizures	14.3	ILAE	LCM(mean dose:323.5±66.9mg/d)	/	15.3±11.7	28.7%	16.2%	NR
Maloney 2018	Retrospective cohort study	T:39<br>C:38	T:23(9)<br>C:7(4)	NR<br>NR	Ireland	Focal epilepsy:36; Generalised epilepsy:4; Genetic generalised epilepsy:1; Unclassified epilepsy:4	1	ILAE	LCM(mean dose:300mg/d, LCM monotherapy)	LCM:(300mg/d, Failed LCM monotherapy)	12	10.0%	6.6%	0
Neal 2018	Ambispective cohort study	NR	310(142)	NR	Australia	Focal-onset seizures	21.3±14.0	ILAE	LCM(initial dose:50-100mg/d; maximum dose:200mg/d; increased 50-100md a week)	/	12	44.5%	11.2%	NR

Villanueva 2018	Retrospective cohort study	T:<65 C:≥65	439 T:311 C:128	NR	Spain	Focal-onset seizures	≥16	ILAE	LCM(Median dose in first-line monotherapy: 200mg/d (50-400mg/d); Median dose in conversion to monotherapy patients: 300mg/d (50-600mg/d))	LCM(Median dose in first-line monotherapy: 200mg/d (50-400mg/d); Median dose in conversion to monotherapy patients: 300mg/d (50-600mg/d))	12±3	11.8% T:10.0% C:16.4%	1.4% T:1.3% C:1.6%	0 T:0 C:0
Sarkis 2017	Retrospective cohort study	T:>60 C:>60	T:71(26) C:39(13)	Caucasia; Other	US	T:Focal epilepsy:94%, Medically refractory:49%; Generalized tonic clonic seizures:43.4% C:Focal epilepsy:85%; Medically refractory:46%; Generalized tonic clonic seizures43.6%	T:14.6±19.5 C:24±21.4	NR	LCM(initial dose:100mg/d; maximum dose:200mg/d; gradually increased)	ZNS(Initial dose:100mg/d; maximum dose:200mg/d; gradually increased)	T:23.1±21.2 C:46.3±38.3	T:38% C:51%	T:27% C:28%	NR
Kurth 2016	Retrospective cohort study	T:43.4±12.1(19-70) C:38.9±14.1(15-71)	T:70(36) C:70(29)	NR	Germany	Refractory Status Epilepticus	T:28.5 C:24	NR	LCM(Mean dose:374.5mg/d(150-600mg/d)	PER(Mean dose:8.6mg/d(4-14mg/d)	3–6	T:45.7% C:72.9%	NR	NR
McGinnis 2016	Retrospective cohort study	<21	223(119)	White; Black; Asian; Other or unkown	US	Focal:168; Generalized:12; Both focal and generalized:43	NR	ILAE	LCM(mean maximum dose:6.6(1.4-25.1)mg/kg/day)	/	7.4(1-53)	42%	21.5%	NR
Giraldez 2015	Retrospective cohort study	49.4(16-92)	66(29)	NR	Spain	Focal-onset seizures	6.5±9.9	NR	LCM(mean dose:270.8mg/d,qd)	/	0.5–54	22.70%	1.52%	0
Zadeh 2015	Prospective cohort study	NR	456(233)	NR	Austria; Bulgaria; Czech Republic; DenmarkFinland; France; Greece, Italy; Romania; Russia,	Simple partial; Complex partial; Generalised seizures	NR	ILAE	LCM(initial dose:100mg/d; maximum dose 400mg/d; increased by 100 mg/day/week)	/	NR	72.8%	2.3%	5.5%

					Spain; Turkey; Mexico; Us									
Brodie 2014	Prospective cohort study	T:42(14-74)<br>C1:42(18-75)<br>C2:40(16-78)<br>C3:42(15-80)<br>C4:44(18-76)	T:160(74)<br>C1：136(65)<br>C2:141(63)<br>C3:135(73)<br>C4:160(74)	NR	UK	Focal-onset seizures	NR	ILAE	LCM(initial dose:50mg/d; maximum dose:200-400mg/d; gradually increased)	C1:TPM(Initial dose:25mg/d; maximum:100mg/d; gradually increased)<br>C2:LEV(Initial dose:500-1000mg/d; Then:250–500mg/d)<br>C3:ZNS(Initial dose:50mg/d; maximum dose:300-500mg/d; gradually increased)<br>C4:PGB(Initial dose:75mg/d; maximum dose:150mg/d; gradually increased)	NR	T:22.5%<br>C1:31.1%<br>C2:32.4%<br>C3:41.8%<br>C4:50.4%	NR	0
Verrotti 2013*	Prospective cohort study	T:10.8±3.14（4-15）<br>C:21.0±5.62(16–38)	T:59(30)<br>C:59(34)	NR	Italian; Germany	Generalize; Focal; Mixed	NR	ILAE	LCM(initial dose:1mg/kg/d; maximum dose:3-12mg/d; gradually increased)	LCM(Initial dose:100mg/day;Maximum dose:100-600mg/d)	12	T:30.5%<br>C:28.8%	T:3.4%<br>C:8.5%	NR
Fountain 2012	Prospective cohort study	39.0±11.66<br>T1:39.1±11.77<br>T2:38.6±11.53<br>T3:39.6±12.24	100(51)<br>T1:25(11)<br>T2:50(24)<br>T3:25(16)	White; Black; Other	US	Simple partial; Complex partial seizures with or without secondary general	23.2±13.97<br>T1:20.0±12.16<br>T2:23.0±13.54<br>T3:26.8±16.08	NR	T1:LCM(200mg/d,iv)<br>T2:LCM(300mg/d,iv)<br>T3:LCM(400mg/d,iv)	/	NR	79%	T1:0<br>T2:6%<br>T3:16%	<5%
Villanueva 2012	Retrospective cohort study	42.1±15.3(12-83)	158(82)	NR	Spain	Focal-onset seizures	23.5±15.3	ILAE, 1981	LCM(initial dose:100-200mg/d; maximum dose:400mg/d; increases of 50 mg qd or 50 mg bid weekly)	/	12	49.4%	19%	0
Zhao 2021*	Case series	7.2±3.9	72(44)	Asian	China	Generalized seizure:27(30%); Focal seizure:25(35%); Complex seizures:20(28%)	3.1±2.7	ILAE,2017	LCM(maintenance dose:6.8mg/kg/d (1.6-20mg/kg/d))	/	12	30.5%	1.4%	0

Ghassan 2020*	Case series	10(2-18)	58(41)	NR	Lebanon	Focal-onset seizures	NR	NR	LCM(initial dose: 2mg/kg/d; maximum dose:10mg/kg/d; weekly increments of 2mg/kg/d))	/	24	29.3%	3.4%	NR
Ruda 2020	Case series	44.5±14.7(16-83)	93(50)	NR	Italy; France; United Kingdom; German; Netherlands; Spain	Focal-onset seizures	NR	NR	LCM(mean dose: 220.7±94.9mg/d)	/	6	16.1%	5.4%	1.1%
Kleist 2019	Case series	36.2±12.9(18-63)	80(51)	NR	Germany	Refractory focal or generalised epilepsy	27.9(3-59)	ILAE	LCM(initial dose:50-100mg/day; mean maximum dose：344.7mg/day(100-700mg/day))	/	21-24	37.5%	14.0%	NR
Ruegger 2019*	Case series	<16	107(61)	NR	Australia	Focal:57(53%); Generalised:50(47%)	NR	NR	NR	/	12	13.1%	6.7%	NR
Baker 2018	Case series	41±12.8(16-82.5)	165(101)	NR	US	Focal epilepsy:146(88.4%); Idiopathic generalized epilepsy:13(7.9%); Symptomatic (structural); Generalized epilepsy:4(2.4%); Classified seizures:2(1.3%)	27.6	NR	LCM(maintenance dose:<200mg:54(32.7%);200-300mg:16(9.7%); 300-400mg:48(29.%),> 400mg:47(28.4%))	/	31.2(3-54)	42.4%	27.8%	NR
Francesc 2018*	Case series	<18	191(104)	NR	Spain	Tonic:92(48.2%); Complex partial:83(43.5%); Simple partial:66(34.6%); Tonic–clonic, generalized:26(13.6%); Epileptic spasms:25(13.1%); Partial secondarily generalized:22(11.5%); Myoclonic:18(9.4%); Hypomotor:5(2.6%); Gelastic:2(1.0%)	5.4±3.7	NR	LCM(mean dose:6.7mg/kg/d(1.3-13.0mg/kg/d))	/	12	28.8%	3.7%	NR

						Atypical absence:1(0.5%); Typical,absence:1(0.5%); Startle:1(0.5%)								
Maher 2018	Case series	30.9(12-65)	104(47)	NR	Lebanon	Focal partial-onset seizures	NR	NR	LCM(initial dose:200mg/d; maximum dose:600mg/d; gradually increased)	/	13.6(2-34)	31.7%	2.0%	0
Munoz-Lopetegi 2018	Case series	50.3(15-88)	73(36)	NR	Spain	Focal seizure:40(54.8%)	3 (0–65)	ILAE	LCM(mean dose:211.4mg/d(100-400mg/d))	/	12	20.5%	11.1%	NR
Ngampoopun 2018*	Case series	11(7-16)	11	NR	Tailand	Nonconvulsive Status Epilepticus:2(18.2%); Acute repetitive seizures:9(81.8%)	0.25	NR	LCM(initial dose: 10mg/kg/d; maximum dose:400mg/d; gradually increased))	/	0.25	9.1%	0	NR
Rosati 2018*	Case series	10.6±4.8	88(47)	NR	Italy	Focal:57(64.8%); Generalized:5(9.3%); Combined:13(24.1%)	6.8±4.6	ILAE	LCM(initial dose:2-3mg/kd/d; maximum dose:5-10mg/kg/d; gradually increase every week)	/	72	12.5%	NR	NR
Rudà 2018	Case series	48.6±14.9	71(48)	NR	Italy	Gliomas and uncontrolled seizures	NR	NR	LCM(initial dose:100mg/day; maximum dose:400mg/day; increase 50mg per week)	/	9	12.7%	NR	NR
Samarasekera 2018	Case series	21-54	8(5)	NR	Australia	Refractory sleep-related hypermotor epilepsy	NR	NR	LCM(mean dose:400mg/d(300–600 mg/d))	/	21.5	50.0%	NR	NR
Toledo 2018	Case series	T:19-56 C:27-81	T:16 C:32	NR	Spain	Cancer-related seizures	NR	NR	LCM(Initial dose:200mg/day(100-400mg/day); maximum dose:400mg/day(200-600mg/day); gradually increased)	LCM(Initial dose:200mg/day(100-600mg/day); Maintenance dose:300mg/day(100-800mg/day); gradually increased)	30.5	75.0%	NR	0

Baulac 2017	Case series	39.7±12.6	120(46)	American Indian/Alaskan Native; Asian; Black; White; Other/mixed	Australia	Focal seizures:120(100%); Simple focal with motor symptoms:42(35.0%); Simple focal with somatosensory symptoms:12(10.0%); Simple focal with autonomic symptoms:5(4.2%); Simple focal with psychic symptoms:7(5.8%); Complex focal:90(75.0%); Focal evolving to secondarily generalized:82(68.3%)	19.89±15.60	NR	LCM(initial dose:100mg/d; maximum dose:200-600mg/d; gradually increased)	/	5.25	75.0%	6.7%	3.0%
Bottcher 2017	Case series	32.7±14.4(2-66)	136(74)	NR	Germany	Focal-onset seizures:96%	26.6±15.1	ILAE	LCM(mean ±SD dose:411.0±119.1mg(100-800mg/d))	/	6	11.0%	11.0%	NR
Brenner 2017	Case series	41.7±15.0(18-78)	132(75)	NR	Netherlands	Focal onset:4(3%); (Multi)focal onset with secondary generalization:77 (58.3%) Generalized onset:31(23.5%) Unknown:20(15.2%)	NR	ILAE	LCM(mean ±SD dose:243.7±104.9mg/d; 50.0-450.0mg/d)	/	4.6±2.1(0.1-7.7)	62.9%	10.6%	NR
Rinesalo 2017	Case series	68(61-84)	67(37)	NR	Finland	NR	8.8(0.9-60)	NR	LCM(mean dose:368 mg/d(100-600mg/d))	/	16(1.5-48)	33.9%	5.6%	0
Sanchez 2017	Case series	54.1±13.8	39(36)	NR	Spain	Secondary epileptic seizures	NR	NR	LCM(mean dose:138.5±68.3 mg/d)	/	6	12.0%	4.0%	0
Svendsen 2017	Case series	39(4–86)	344(165)	NR	Norway	SE,ARSs	NR	NR	LCM(mean dose:299mg/d)	/	NR	44.1%	7.0%	0

Arkilo 2016*	Case series	6.5(0.6-12)	47	NR	US	Rpilepsia partialis continua; Status epilepticus; Acute seizure exacerbation	NR	NR	Epilepsia partialis continua:LCM(5-10mg/kg) Status epilepticus:LCM(median dose 7.2mg/kg, range 4-11mg/kg) Acute exacerbation of seizure frequency (median dose 4.5mg/kg, range 1-11mg/kg)	/	NR	13.5%	NR	0
Borzì 2016	Case series	42.7±12.11 (20-71)	86(36)	NR	Italy,Germany	Temporal lobe epilepsy	NR	ILAE	LCM:initial dose:50mg/day, maximum dose:200-400mg/day, increased by every 50mg weekly	/	18(6-46)	3.5%	NR	NR
Miskin 2016	Case series	11.9(4-19.5)	21(10)	NR	US	Refractory generalized epilepsy	NR	NR	LCM(initial dose:2.9mg/kg/d(0.8-5.9mg/kg/d); maximum dose:6.9mg/kg/d(1.7-14.3mg/kg/d); gradually increased)	/	19(3-43.3)	28.6%	4.8%	NR
Vossler 2016	Case series	40.7 ±13.3(16-69)	322(161)	White：80.1%	US; Canada; Europe; Australia	Focal-onset seizures	16.40±13.56	NR	LCM(median dose:500mg/d(150-800 mg/d))	/	24	91.0%	6.8%	NR
Geffrey 2015	Case series	20±10(1-54)	46(21)	NR	US	Refractory epilepsies	12	NR	LCM(Mean dose:7±3mg/kg/d; In children:po 1.42-15.69mg/kg/d; In adults:po 2.09-8.90mg/kg/d)	/	20±20	32.6%	NR	NR
IJff 2015	Case series	37.0±14.5(16-74)	32(9)	NR	Netherlands	Refractory epilepsy	NR	NR	LCM(initial dose:50mg/d; maximum doseL400mg/d; increased 50mg/d per week)	/	7±6(1-24)	9.1%	9.1%	NR
Lattanzi 2015	Case series	40.5(28-47)	58(26)	Caucasian	NR	Focal-onset seizures	NR	ILAE	LCM(maintenance dose:400mg/d)	/	12	20.7%	NR	0

René 2015	Case series	27.1±14.2(18-61)	19(12)	NR	Colombia	Atonic; Tonic; Tonic-clonic seizures	22.5(5-59)	ILAE	LCM(initial dose:25-50mg/d(median 25mg/d); maximum dose:200mg/d;gradually increased every 2 weeks)	/	18	47.4%	NR	NR
Runge 2015	Case series	47.3±17.0	571(283)	NR	Germany	Focal-onset seizures	8.0 (0–64)	NR	LCM(mean±SD dose：279.2±101.3mg/d)	/	6	48.5%	10.7%	9.80%
Garcés 2014	Case series	59.8(18–91)	98(42)	NR	Spain	NCSE/CPS:56(57.1%); NCSE/CPS:56(57.1%); CSE(motor SPS)/motor SPS:17(17.3%)	18.0±17.6	ILAE	SE:LCM(mean loading dose:190.9mg/d; mean maintenance dose:217.7mg/d) ARS:LCM(mean loading dose:187.2mg/d; mean maintenance dose:202.3mg/d)	/	NR	15.6%	NR	NR
Grosso 2014*	Case series	2.7±1.1(1.9-3.9)	24(14)	NR	Italy	Refractory focal epilepsy	NR	ILAE	LCM(initial dose:1-2mg/kg/d; max dose:7-15.5mg/kg/d; gradually increased)	/	3	33.3%	NR	0
Kim 2014*	Case series	13.9(1.2-17.9)	21(16)	NR	Koera	Refractory focal epilepsy	4.1(1.0-14.4)	NR	LCM(mean maintenance dose:5.4mg/kg/d(1.4-9.8mg/kg/d))	/	10.1(6.1–13.0)	38.1%	10.0%	NR
Stephen 2014	Case series	42(14-71)	160(74)	NR	UK	Focal-onset seizures	NR	NR	LCM(initial dose:50mg/d; maximum dose:200-400md/d; gradually increased)	/	24	NR	15.0%	NR
Villanueva 2014	Case series	44.9(16-88)	199(104)	NR	Switzerland	Frontal 45 (22.7%); Temporal 98 (49.5%); Parietal 12 (6.1%); Occipital 9 (4.5%); Lobe unknown 34 (17.2%)	NR	NR	LCM(mean±SD dose:291±103.6mg/d(median 300mg/d))	/	12	NR	8.3%	NR
William 2014	Case series	40.8±11.01	370(178)	Whith; Black; Asian； Other	Germany; Hungary; Lithuania; Poland; Sweden; Switzerland; UK; US	Focal-onset seizures	25.3±12.55	NR	LCM(100mg/d:2.2%; 200mg/d:11.6%; 300mg/d:15.4%; 400mg/d:22.2%; 500mg/d:15.9%; 600mg/d:21.9%; >600mg/d:10.8%)	/	12	33.8%	13.2%	NR

Giorgi 2013	Case series	48.2±14.8(26-76)	10(6)	NR	Italy	Focal limbic seizures, SG; Nocturnal frontal seizures	NR	ILAE	LCM(initial dose:100mg/d; maximum dose:250±81.6mg/d; weekly increments of 50 mg/d)	/	NR	60.0%	0	NR
Li 2013	Case series	40.2 ± 10.9	17(7)	Caucasian	Germany	Pharmacoresistant focal epilepsy	19	NR	LCM(iv.,200mg,q12h,2-3d; then po,200mg,q12h,2-3d)	/	3	17.6%	NR	NR
Villanueva 2013	Case series	42.4(18-88)	500(268)	NR	Spain	Focal-onset seizures	1.79±1.19	ILAE	LCM(mean±SD dose:338.5±96.3mg/d; median dose:400mg/d)	/	12	NR	9.0%	NR
Carlos 2012*	Case series	8.01-4.25(0.5-16)	130(72)	NR	Spain	Simple:32(24.6%); Complex:93(71.5%); Secondarily generalized:84(64.6%)	NR	ILAE	LCM(initial dose:1-2mg/kg/day; maximum dose:6-9mg/kg/day; gradually uptitrated)	/	12	30.0%	2.3%	NR
Eli 2012*	Case series	8±4.7(1.5-16)	17(10)	NR	Israel	Dyscognitive:17; Evolving to a bilateral convulsive:3; Tonic:4; Tonic Atonic:1; Generalized tonic-clonic:2	5.4±3.3	NR	LCM(initial dose:1.4-5mg/kg/d(average:3.04±1.09mg/kg/d); maximum dose:6.7-20mg/kg/d(average:12.39±4.48mg/kg/d); gradually increased)	/	9.1±4.4	58.8%	NR	NR
Flores 2012	Case series	41.9(17-82)	285(148)	NR	UK	Tonic clonic seizures:107; Complex partial seizures:221; Simple partial seizures:34	NR	NR	Initial dose: po,25-200mg qd; most frequent dose:50mg/d; mean maximum dose:po,279.4(25-700) mg qd; orally	/	11.6(0.1-42)	47.9%	20.8%	NR
Harden 2012	Case series	38(18-82)	67(32)	NR	US	Complex partial:61(91%); Generalized tonic clonic:24(36%); Atonic:11(16%)	22	NR	LCM(mean dose:278mg/d(50-600mg/d))	/	7(1-12)	55.2%	7.5%	0
Husain 2012	Case series	38.2±12.46	308(162)	NR	US	Focal-onset seizures	23.8±12.97	NR	LCM(mean dose:200mg/d(100-800 mg/d); increased 100mg/d a week)	/	12	93.5%	11.4%	23.1%

Kamel 2012	Case series	18–72	128(64)	NR	Australia	Focal-onset seizures	NR	NR	LCM(initial dose:100mg/d; mean dose:250mg/d)	/	18	40.6%	19.0%	NR
Song 2012*	Case series	8±4.7(1.5-16)	17(10)	Asian	China	Partial epileptic seizure:12; Partial seizures secondary to generalized seizure:3; Generalized epileptic seizure:2	5.4±3.3	NR	LCM(initial dose:1.4-5mg/kg bid(average:3.04±1.09 mg/kg/d); maximum dose:6.7-20mg/kg qd(average:12.39±4.48 mg/kg/d); gradually increased)	/	9.1±4.4(1-18)	58.8%	11.8%	NR
Yorns 2012	Case series	13.1±4.9(<21)	40(22)	NR	US	Generalized tonic clonic:36(88%); Atonic:12(30%); Tonic:11(27.5%); Myoclonic:10(25%); Simple partial:6(15%); Complex partial:30(75%); Absence:15(37.5%); Multiple types:28(70%).	NR	NR	LCM(initial dose:2.0±0.7mg/kg/d. maximum dose:7.0±4.2mg/kg/d; gradually increase)	/	9.2±4.7	37.5%	7.5%	NR
García-Morales 2011	Case series	38.3(21–69)	60(28)	NR	Spain	Refractory focal epilepsy, nocturnal seizures	27.2(1-67)	ILAE	LCM(initial dose:50mg/d; maximum dose:200-500mg/d; weekly increments of 50mg/d)	/	13-24	33.3%	10.0%	0
Gavatha 2011*	Case series	10.58±7.58(3-18)	18(10)	NR	Greece	Focal-onset seizures	NR	NR	LCM(initial dose:1mg/kg/d; maximum dose:6.34mg/kg/d(1.7-10mg/kg/d);increased 1mg/kg/week)	/	12	38.9%	5.6%	NR
Giorgi 2011	Case series	48.2±14.8(26-76)	10(6)	NR	Italy	Focal symptomatic epilepsy:5; Probably focal symptomatic epilepsy:5	13.5±7.9	ILAE	LCM(initial dose:50mg/d; maximum dose:250±81.6mg/d; gradually increased)	/	6	60.0%	0	0
Guilhoto 2011	Case series	14.9±3.3(8–21)	16(7)	NR	Brazil	Drug-resistant focal epilepsy	NR	ILAE	LCM(initial dose:50mg/d(71.8±25.6 mg/d); maximum dose:275mg/d(262.5±158.6mg/d); gradually increased)	/	4(1-13)	NR	25.0%	NR

Julia 2011	Case series	62(17-95)	48(22)	NR	Austria	Seizure cluster:17; Status epilepticus:31	NR	NR	LCM(initial dose:200mg/d(200-400 mg/d),iv.; loading dose:0(0-400mg),iv)	/	NR	4.2%	NR	NR
Maschio 2011	Case series	22-63	14(8)	NR	Italy	Brain tumor-related epilepsy	NR	ILAE	LCM(initial dose:100mg/d; maximum dose:400mg/d; gradually increased; mean dose:332.1mg/day(100-400mg/day))	/	5.4(<1-10)	NR	7.14%	NR
Kellinghaus 2010	Case series	62.64±19.7	39(18)	NR	Germany	Generalized convulsive:6; Complex partial:17; Simple partial:16	NR	NR	LCM (median dose:400mg/d(200-400 mg/d))	/	NR	NR	NR	0
Stamm B 2020	Case report	32	1(1)	NR	NR	Drug-resistant epilepsy with focal impaired awareness seizures	NR	NR	LCM(400mg/d i.v.)	/	NR	NR	NR	NR
Lachuer 2012	Case report	88	1(0)	NR	France	Epileptic syndrome	NR	NR	LCM(100mg/d i.v.)	/	NR	NR	NR	NR
Krause 2011	Case report	89	1(0)	NR	Germany	Nonconvulsive status epilepticus	NR	NR	LCM(400mg i.v. 2 times within 6 hours)	/	NR	NR	NR	NR

LCM: Lacosamide; TPM: Topiramate; ZNS:Zonisamide; LEV:Levetiracetam; PGB:pregabalin; PER: Perampanel; CBZ: Carbamazepine.

RCT: Randomized clinical trial.

NR: Not reported.

Study*: Studies which only included children under the age of 18.

Supplementary table 2 Quality evaluation of includes studies

RCT(n=11)				Cohort study(n=16)		Case series(n=54)		Case report(n=3)	
**Cochrane**	**high risk**	**low risk**	**unclear**	**NOS**		**NICE**		**CARE**	
Item 1	0(0%)	4(36.4%)	7(63.6%)	selection		Item 1	12(22.2%)	Item 1	3(100%)
Item 2	1(9.1%)	3(27.3%)	7(63.6%)	Item 1	14(87.5%)	Item 2	53(98.1%)	Item 2	3(100%)
Item 3	1(9.1%)	3(27.3%)	7(63.6%)	Item 2	14(87.5%)	Item 3	46(85.2%)	Item 3	3(100%)
Item 4	2(18.2%)	3(27.3%)	6(54.5%)	Item 3	8(50%)	Item 4	43(79.6%)	Item 4	3(100%)
Item 5	1(9.1%)	9(81.8%)	1(9.1%)	Item 4	16(100%)	Item 5	45(83.3%)	Item 5	2(66.7%)
Item 6	0(0%)	9(81.8%)	2(18.2%)	comparability		Item 6	39(72.2%)	Item 6	3(100%)
Item 7	0(0%)	1(9.1%)	10(90.1%)	Item 1	16(100%)	Item 7	49(90.7%)	Item 7	3(100%)
				Item 2	7(43.8%)	Item 8	6(11.1%)	Item 8	2(66.7%)
				outcome		mean score	5.4	Item 9	3(100%)
				Item 1	6(37.5%)			Item 10	2(66.7%)
				Item 2	12(75%)			Item 11	3(100%)
				Item 3	9(56.3%)			Item 12	1(33.3%)
				mean score	6.3			Item 13	1(33.3%)

Yes=1, No=0.

RCTs: item 1 Random sequence generation; item 2 Allocation concealment; item 3 Blinding of participants; item 4 Blinding of outcome assessment; item 5 Incomplete outcome data; item 6 Selective reporting; item 7 Other sources of bias.

Cohort study: selection: item 1 Representativeness of the exposed cohort; item 2 Selection of the non exposed cohort; item 3 Ascertainment of exposure; item 4 Demonstration that outcome of interest was not present at start of study; Comparability: item 1 Study controls for the most important factor; item 2 Study controls for any additional factor; Outcome: item 2 Assessment of outcome; item 2 Was follow-up long enough for outcomes to occur; item 3 Adequacy of follow up of cohorts.

Case series: item 1 Case series in more than one center, that is, multicenter study; item 2 Is the hypothesis/aim/objective of the study clearly described? item 3 Are the inclusion and exclusion criteria (case definition) clearly reported? item 4 Is there a clear definition of the outcomes reported? item 5 Were data collected prospectively? item 6 Is there an explicit statement that patients were recruited consecutively? item 7 Are the main findings of the study clearly described? item 8 Are outcomes stratified? (e.g., by disease stage, abnormal test results, patient characteristics).

Case reports: item1 Title; item2 Key words; item3 Abstract; item4 Introduction; item5 Information of patient; item6 Clinical findings; item7 Timeline description; item8 Diagnostic evaluation; item9 Treatment and intervention; item10 Follow-up and outcomes; item11 Discussion; item12 Patient's view; item13 Informed Consent.
